# Home-Based Aerobic Interval Training Improves Peak Oxygen Uptake Equal to Residential Cardiac Rehabilitation: A Randomized, Controlled Trial

**DOI:** 10.1371/journal.pone.0041199

**Published:** 2012-07-18

**Authors:** Trine Moholdt, Mona Bekken Vold, Jostein Grimsmo, Stig Arild Slørdahl, Ulrik Wisløff

**Affiliations:** 1 K.G. Jebsen Center of Exercise in Medicine, Norwegian University of Science and Technology, Department of Circulation and Medical Imaging, Trondheim, Norway; 2 Department of Public Health, Norwegian University of Science and Technology, Trondheim, Norway; 3 Department of Cardiac Rehabilitation, Feiringklinikken (The Feiring Heart Clinic), Feiring, Norway; 4 Department of Cardiology, St Olav’s Hospital, Trondheim, Norway & Department of Circulation and Medical Imaging, Norwegian University of Science and Technology, Trondheim, Norway; University of Virginia Health System, United States of America

## Abstract

Aerobic capacity, measured as the peak oxygen uptake, is a strong predictor of survival in cardiac patients. Aerobic interval training (AIT), walking/running four times four minutes at 85–95% of peak heart rate, has proven to be effective in increasing peak oxygen uptake in coronary heart disease patients. As some patients do not attend organized rehabilitation programs, home-based exercise should be an alternative. We investigated whether AIT could be performed effectively at home, and compared the effects on peak oxygen uptake with that observed after a standard care, four-week residential rehabilitation. Thirty patients undergoing coronary artery bypass surgery were randomized to residential rehabilitation or home-based AIT. At six months follow-up, peak oxygen uptake increased 4.6 (±2.7) and 3.9 (±3.6) mL·kg^−1^ min^−1^ (both p<0.005, non-significant between-group difference) after residential rehabilitation and AIT, respectively. Quality of life increased significantly in both groups, with no statistical significant difference between groups. We found no evidence for a different treatment effect between patients randomized to home-based AIT compared to patients attending organized rehabilitation (95% confidence interval −1.8, 3.5). AIT patients reported good adherence to exercise training. Even though these first data indicate positive effects of home-based AIT in patients undergoing coronary artery bypass surgery, more studies are needed to provide supporting evidence for the application of this rehabilitation strategy.

**Trial Registration:**

ClinicalTrials.gov NCT00363922

## Introduction

Cardiac rehabilitation programs including moderate intensity exercise have in meta-analyses been found to prevent mortality in coronary heart disease patients [1]. Higher exercise intensities elicit larger improvements in peak oxygen uptake (VO_2peak_) in healthy subjects [2], [3]. Although some studies indicate otherwise [4], [5], several recent randomized controlled trials have confirmed this also in coronary heart disease (CHD) patients [6]–[9]. We have used aerobic interval training (AIT) with exercise intensity at about 90% of individual heart rate maximum for four minutes, repeated four times, to improve VO_2peak_ and left ventricular function in CHD patients [4], [6]–[8]. One criticism of high intensity training has been that it is not feasible for patients to do it without supervision. As many cardiac patients who could benefit from exercise training are not included in organized exercise training programs [10], we were interested in studying the effects and feasibility of home-based interval training with high intensity in CHD patients. Furthermore, home-based forms of rehabilitation has previously been found to be equally effective in improving clinical and health related quality of life in cardiac patients [11]. In former studies, the exercise intensity has been moderate, and the adherence to higher intensity exercise training in a home setting was unknown. The clinical question we were asking was therefore whether home-based AIT could be as effective as residential rehabilitation after coronary artery bypass surgery. The primary aim of our study was to compare changes in VO_2peak_ after home-based AIT with the ones seen after a standard four week residential rehabilitation program. Our hypothesis was that patients receiving residential rehabilitation would have a higher increase in VO_2peak_ at the follow-up testing compared to the home-based AIT group due to insufficient exercise adherence in the home-based group. A secondary aim of the study was to investigate the feasibility of AIT in a home setting after coronary artery bypass surgery.

## Materials and Methods

The protocol for this trial and supporting CONSORT checklist are available as supporting information; see Checklist S1 and Protocol S1. The study was approved by the Regional Committee for Medical and Health Research (REC, Norway). Informed, written consent was obtained and all clinical investigation was conducted according to the principles expressed in the Declaration of Helsinki. The trial design was a randomised controlled trail with 1∶1 allocation to parallel groups. Thirty patients undergoing coronary artery bypass surgery (63±7.7 years, 6 women) were randomised after surgery to a four week residential rehabilitation program or home-based AIT. Patients were eligible if they went through coronary artery bypass surgery four to eight weeks ago and were clinically stable (defined as the absence of unstable angina pectoris, symptoms of heart failure, pleural liquid limiting respiration, lung disease limiting respiration, on-going infections, and atrial fibrillation limiting circulation). Exclusion criteria were left ventricular ejection fraction <30%, contraindications to vigorous physical activity (unstable angina, uncontrolled abnormal heart rhythms, severe aortic stenosis, suspected or known dissecting aneurysm, infection in the heart or any other systemic infection), pulmonary disease clearly limiting exercise capacity, pregnancy, or drug abuse. Data were collected at the Feiring Heart Clinic, Feiring, Norway. The four week residential rehabilitation program was a standard program at the Feiring Heart Clinic rehabilitation centre, and can be regarded as usual care for these patients in Norway. Initially, we had planned to include patients also from another hospital, and to compare residential cardiac rehabilitation with policlinic rehabilitation for the patients recruited at the second hospital. Our sample size estimation was therefore done with regard to also this comparison. With a statistical power of 0.8 (p<0.05) and an expected group difference in improvement in peak oxygen uptake of 3.0 mL/kg/min (±4.0), we estimated that we would have to include 60 patients in total, 30 at each site. As we experienced difficulty in conducting the study at one of the sites, we decided to include only 30 patients to test the hypothesis of the present study.

Exercise intensity was set using the Borg 6–20 scale of perceived exertion [12], with light intensity as up to 11 on the scale, moderate intensity as 12–14, and high intensity as 15–17. During the stay the patients did 30 exercise sessions; four with low intensity, 16 with moderate intensity, and ten with high intensity. The activities included outdoor walking, cross-country skiing in winter time, indoor cycling, ball games, and strength training. The main focus was endurance type exercise training (80% of sessions). The rehabilitation program also included diet counselling, a smoking cessation program, and lectures about healthy lifestyle in general. After discharge from the rehabilitation centre, the patients were advised to keep on exercising at home, and were invited back for follow-up testing after six months. The residential group did not receive a training diary and concrete advice about how to exercise at discharge, as this is standard care at the rehabilitation centre. They were however encouraged to continue exercising at home.

Patients randomised to home-based AIT received oral instructions in how to do AIT. They were offered 60 minutes of theoretical background for why high intensity training is effective in increasing physical capacity, and practical guidelines in how to do this on their own. They were asked to do the AIT program in the home setting three times per week for a period of six months. In each training session, they should warm up for ten minutes, followed by four intervals of four minutes of high intensity exercise. In these four minutes they should breathe heavily without pain in legs or chest, and with heart rates of 85–95% of individual maximum. After each interval, they should exercise with moderate intensity for three minutes, at heart rate of about 70% of individual maximum. The total exercise time of an AIT session was therefore 38 minutes, of which 16 minutes was high intensity exercise. They were allowed to choose activities that they liked (using large muscle groups), like walking, jogging, swimming or cycling. Regarding safety issues when exercising at home, they were told to contact either the rehabilitation centre staff or their general practitioner if they experienced any symptoms during or after exercising. They got written guidelines describing the AIT program and also about healthy lifestyle, and a training diary. Both groups came back for follow-up testing after six months.

Primary outcome measure was peak oxygen uptake (VO_2peak_) tested on treadmill. Respiratory gas was analysed and a 12-lead electrocardiogram was monitored continuously. We used ramp protocols, individually adjusted to last 8–12 minutes [13]. Reasons to stop were subjective exhaustion or standard clinical criteria [13]. We asked the subjects of their perception of exhaustion immediately after ending the test using the Borg 6–20 scale [12]. We measured heart rate recovery as the change in heart rate from peak exercise to one minute after peak exercise with the patient standing [14]. Heart rate recovery has been found to be an independent predictor of survival in cardiac patients [15].

Secondary outcomes were quality of life, serum levels of glucose, glycated haemoglobin, ferritin, total- and high-density lipoprotein (HDL) cholesterol, and triglycerides. Venous blood was drawn after a 10-hour overnight fast. Blood lipids and glucose were analysed using Vitros 350 (Johnson & Johnson AB, Sweden), ferritin was analysed using Vitros ECi (Johnson & Johnson AB, Sweden), and glycated haemoglobin using DCA 2000 (Siemens Medical Solutions Diagnostics, Norway), Quality of life was measured by the heart specific MacNew questionnaire [16]. Also, endothelial function was set as an outcome measure in the original protocol of the study, measured as flow-mediated dilatation of the brachial artery and using blood markers of endothelial function. This outcome measure was not obtained as we experienced practical difficulties in conducting the data collection.

Allocation was done by a computer using block randomisation. The first, the smallest and the largest block, were defined by the technicians at the unit of Applied Clinical Research at the university. The person including the patients got the allocation results on screen and by e-mail by logging on to a website.

The primary analysis of this study was changes in VO_2peak_ between the two groups. Secondary analyses were changes in VO_2peak_ within groups, as well as changes in secondary outcome measures between and within groups. The mean change in each group from baseline to follow-up testing was reported as the estimated margin of the mean (EMM), with 95% confidence intervals (CI). Within-groups differences were considered significant when the 95% CI did not include zero [17]. To test for differences in the changes of the outcome variable from baseline to follow-up testing, we did covariance analyses (ANCOVA). Intervention group was set as a fixed factor and baseline values of the outcome variable as covariates [18]. Tests were two-sided and p-values at or below 0.05 were considered significant. We applied no corrections for multiple tests.

## Results

**Figure 1:**
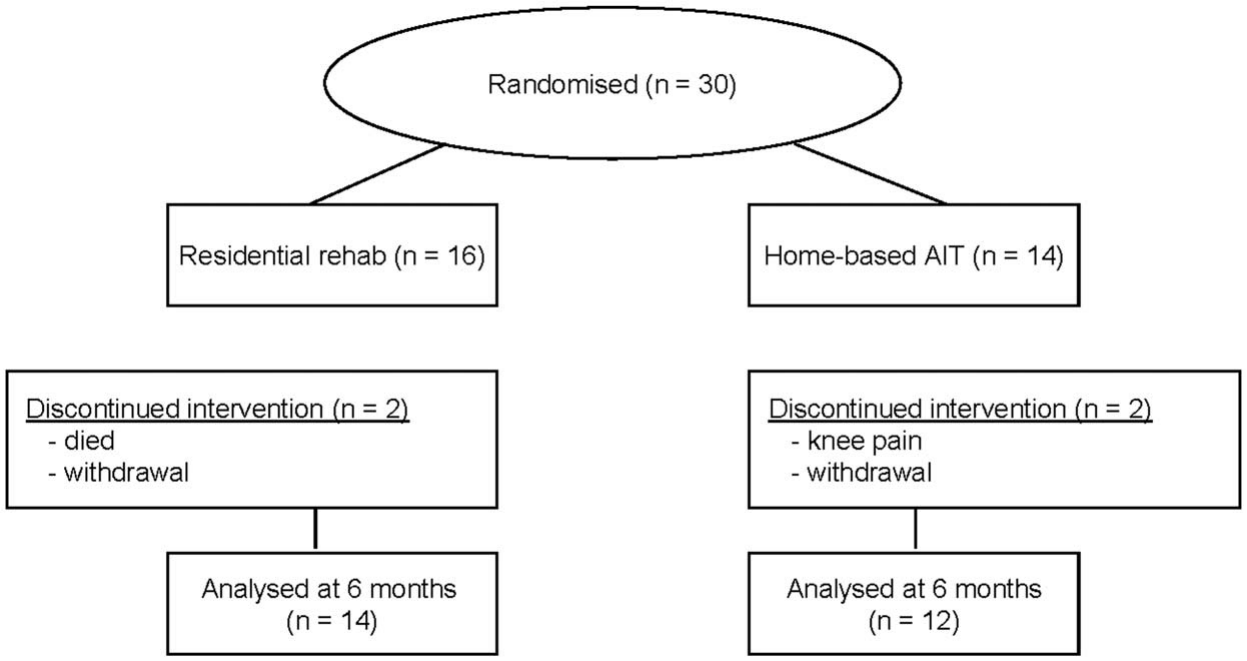
Flow-chart of participants in the study.

Baseline characteristics of the patients are shown in Table 1, and a flow-chart of the study is outlined in Figure 1. We had one adverse event in the study as one patients died during the warm-up of a low intensity skiing session in the residential group. This patients was a 59 year old man with an initial VO_2peak_ of 28.1 mL·kg^−1^ min^−1^ and heart rate recovery of 20. At the baseline test we saw no medical reasons for this patient not to exercise according to the standard rehabilitation program or as home-based AIT. Apart from this event, we experienced no unfortunate side effects of the exercise training programs. Patients were recruited between October 2006 and November 2007, and follow-up tests were done between April 2007 and June 2008.

**Table 1:** Patient characteristics at baseline.

	Residential rehabilitation, n = 16	Home-based aerobic interval training, n = 14
Age, years	63.6±7.3	61.7±8.0
Male/female – no. patients	13/3	11/3
Weeks after coronary artery bypass grafting at inclusion	7.5±1.3	8.0±2.7
Body mass index, kg/m^2^	26.2±3.2	27.5±4.9
Initial peak oxygen uptake, mL kg^−1^ min^−1^	25.1±4.0	24.0±5.7
**Medications – no.patients**		
β-Blockers	15	8
Statins	14	14
Diuretics	2	6
Angiotensin-converting enzyme (ACE) inhibitors	1	1
**Smoking – no. patients**		
Current	0	2
Former	9	9
Never	7	3

Data presented is mean value ± standard deviation if not otherwise stated.

There were no baseline differences between groups in age, gender, weeks after coronary artery bypass surgery at inclusion, body mass index, or initial peak oxygen uptake. More patients in the residential group were taking beta blockers at baseline.

The home exercise and residential group increased peak oxygen uptake with 18.8% and 17.4%, respectively (Table 2), with non-significant between-group difference (ANCOVA, 95% CI (−1.8 to 3.5)). Only the home-based AIT group had significantly higher heart rate recovery at the follow-up, but there was no significant between-group difference in change (ANCOVA, 95% CI (−19.6, 3.8), Table 2). We saw no significant changes in respiratory exchange ratio, body weight, or perceived exertion between baseline and follow-up testing (all 95% CIs included zero, Table 2).

**Table 2:** Outcome variables at baseline and six months follow-up for patients completing follow-up testing.

	Residential rehabilitation (n = 14)				Home-based aerobic interval training (n = 12)		
	**Baseline**	**4 weeks**	**Follow-up**	**EMM (95% CI)**	**Baseline**	**Follow-up**	**EMM (95% CI)**
**Exercise test**							
VO_2_peak (mL·kg-1·min-1)	25.6±4.0	28.5±4.4*	30.2±4.3*	4.7 (2.9, 6.5)	23.8±5.4	27.7±6.5*	3.8 (1.9, 5.7)
VO_2_peak (mL·min-1)	1976±429	2198±493*	2310±513*	335 (189, 481)	2016±555	2387±619*	370 (212, 527)
RER at VO2peak	1.16±0.09	1.19±0.08	1.17±0.06	0.03 (−0.02, 0.08)	1.11±0.06	1.11±0.10	−0.03 (−0.07, 0.02)
HRR, 1 min	21.1±10.3	25.2±9.5	24.8±13.3	4.5 (−2.5, 11.6)	10.9±6.5	24.8±11.5*	12.4 (4.2, 20.6)
Perveived exertion	17±1.2	17.3±1.3	17.5±1.4	0.56 (−0.14, 1.26)	16.5±1.2	16.7±1.3	0.02 (−0.74, 0.77)
**Quality of life**							
Emotional domain	5.8±0.7	–	5.8±1.1	0.01 (−0.40, 0.43)	5.4±0.8	5.6±0.9	0.20 (−0.27, 0.67)
Physical domain	5.5±0.8	–	6.3±0.7*	0.91 (0.57, 1.24)	5.3±0.8	6.2±0.4*	0.83 (0.46, 1.21)
Social domain	5.5±0.8	–	6.3±0.8*	0.84 (0.50, 1.18)	5.2±0.5	5.9±0.6*	0.98 (0.60, 1.37)
**Blood markers**							
HDL, mmol/L	1.1±0.3	–	1.4±0.2*	0.21† (0.11, 0.30)	1.2±0.2	1.2±0.2	0.04 (−0.07, 0.14)
Triglycerides, mmol/L	1.2±0.7	–	1.6±1.1	0.24 (−0.25, 0.73)	1.7±0.6	1.4±0.7	−0.12 (−0.67, 0.43)
Cholesterol, mmol/L	4.2±1.4	–	4.3±1.0	0.19 (−0.23, 0.61)	4.2±0.5	4.3±0.7	0.11 (−0.35, 0.56)
Ferritin, µg/L	153±162	–	101±120*	52 (20, 84)	169±175	116±180*	42 (6, 78)
Glucose, mmol/L	5.5±1.2	–	5.5±0.7	0.02 (−0.34, 0.38)	5.7±0.9	5.8±0.9	0.10 (−0.30, 0.51)
HbA1c	5.3±0.4	–	5.5±0.3*	0.23 (0.05, 0.40)	5.6±0.7	5.9±0.7*	0.28 (0.08, 0.48)

For the residential group, results of exercise tests at 4 weeks are shown. Change scores are between baseline and six months follow-up. If not otherwise stated, values are average ± standard deviation. Quality of life and blood markers were not measured at 4 weeks.

EMM  =  Estimated Marginal Means, 95% CI = 95% Confidence Interval, VO_2peak_  =  Peak oxygen uptake, Perceived exertion is according to the 6–20 Borg scale, RER  =  respiratory exchange ratio, HRR, 1 min  =  heart rate recovery the first minute after ending an exercise test, HDL  =  high density lipoprotein cholesterol. HbA1c  =  glycated haemoglobin.

* Significant change from baseline (within-group difference, *p*<0.05).

† Significantly different change from baseline (between-group difference p<0.05).

Health related quality of life increased significantly within both groups, with non-significant between-group difference (Table 2). We saw significant increases in both the social and the physical domain of the MacNew questionnaire in both groups, but no changes in the emotional domain. The residential group had significantly higher HDL cholesterol at the follow-up, compared to baseline (EMM 0.21, 95% CI (0.11, 0.30)) and to the AIT group (ANCOVA, 95% CI (0.03, 0.31), Table 2). Both groups increased their level of glycated haemogobin between baseline and follow-up testing (Table 2).

Medication at baseline is outlined in Table 1. Two patients in the residential group decreased their β-blocker dosage and one patient quit using ACE inhibitors during the follow-up period. In the home-based AIT group, one patient decreased β-blocker dosage, one quit using β-blockers, and one started using β-blockers between baseline testing and follow-up. There was no change in the number of patients at diuretics during the follow-up period.

Patients in the AIT group registered their training throughout the follow-up period and did 1.6 (±1.6) AIT sessions and 2.4 (±1.9) moderate intensity sessions weekly. Table 3 show individual training amounts and intensities in the AIT group. At least five (two incomplete registrations) of the fourteen patients in the AIT group did the requested AIT three times per week or more during the whole follow-up period. We did not register exercise training after discharge in the residential group as we think that giving patients a training diary will make them do more exercise than they otherwise would have done just following standard care.

**Table 3:** Reported exercise training between discharge from the rehabilitation centre and follow-up testing at 6 months for patients in home-based aerobic interval training group.

	Home-based aerobic interval training, n = 14
**Moderate intensity only**	
<3 times/week moderate intensity, no AIT	1
≥3 times/week moderate intensity, no AIT	4
**High intensity only**	
<3 times/week AIT, no moderate intensity	–
≥3 times/week AIT, no moderate intensity	2
**Both high and moderate intensity**	
<3 times/week AIT + <3 times/week moderate intensity	2
≥3 times/week AIT + <3 times/week moderate intensity	3
**Incomplete registration**	2

Number of patients. AIT  =  Aerobic interval training.

## Discussion

The main finding of our study was significant improvements in VO_2peak_ and quality of life after both residential and home-based AIT in patients undergoing coronary artery bypass surgery. We found no between-group differences in the increase in VO_2peak_ and therefore no evidence for a different treatment effect between the two interventions. We have, however, *no evidence* of equal treatment effects of residential rehabilitation and home-based AIT based on our data. Also others [19] have found similar improvements in VO_2peak_ in hospital versus home-based exercise groups after coronary artery bypass grafting, and a recent Cochrane review found home-based exercise to be as efficient as hospital-based programs [11]. Improvements in quality of life have also earlier been found to be similar between home- versus centre-based trials [20]. New to our study, however, was home exercise specified as AIT, traditionally considered to be difficult to perform without close supervision for cardiac patients. Furthermore, we compared the home-based exercise training with a residential program and not a hospital-based rehabilitation as in most of the earlier studies.

According to training diaries, at least 7 (two incomplete registrations) of the 12 patients reported ≥2 weekly interval sessions the whole 6 month follow-up period. In general, there was an overall acceptable adherence to exercise in the home-based group, making us have to reject the hypothesis of inferior increase in VO_2peak_ in the home-based group due to low adherence to exercise. We found however that some of the patients chose to do moderate exercise instead of AIT, and that some did both moderate exercise and AIT. We do not know of earlier studies investigating home-based AIT in cardiac patients without first attending a residential or policlinic rehabilitation program. However, we have in two previous studies [4], [9] found a quite high adherence rate to home-based AIT after an initial organised program. In both these previous studies, the patients managed to maintain or increase their VO_2peak_ during the follow-up period.

The increase in heart rate recovery (HRR) observed only in the home-based AIT group, was probably caused by a significantly inferior HRR at baseline in this group. The already quite high HRR value at baseline in the residential group is a possible explanation of the lack of improvement in that group. A lower HRR at baseline may indicate that the AIT group was a sicker population than the residential group. However, there was no significant difference in VO_2peak_ at baseline between the two groups, showing that their initial fitness level was similar. Only the residential group improved their levels of HDL cholesterol during the follow-up period, with a significant different change between groups. This was perhaps caused by the diet counselling and practical cooking sessions at the rehabilitation centre received by this group, in contrast to the written material on diet received by the home-based AIT group. The observed significant increase in glycated haemoglobin (HbA1c) in both groups despite participation in cardiac rehabilitation is difficult to explain. We saw no significant change in body weight during the follow-up period, so increased body mass was not the reason for this increase. One possible explanation, however, is that these patients are developing a reduced insulin sensitivity due to an on-going unhealthy lifestyle that also gave them coronary heart disease.

One limitation to our study was the lack of follow-up of elicited exercise intensity and volume after the stay at the rehabilitation centre in the residential group. Due to difficulty in enrolment of patients, our study was under-powered for the primary endpoint and this is also regarded as a limitation. Since our study was not adequately powered, it should serve to stimulate a larger multicentre randomised trial. Our study indicates however, that as little as one hour of instruction in how to do AIT leads to significant improvements in aerobic capacity at the follow-up test (after coronary artery bypass grafting). Furthermore, there is a further need for investigating how home-based exercise training can be arranged as a cost effective alternative to residential cardiac rehabilitation, and to establish the safety aspects of home-based AIT for cardiac populations.

We conclude that both residential rehabilitation and home-based AIT improve VO_2peak_ in patients undergoing coronary artery bypass graft surgery. Further, our study suggests that home-based AIT is a feasible form of training in cardiac patients in a home setting. We have earlier found that patients randomised to AIT after myocardial infarction were able to maintain their VO_2peak_ as long as 30 months after ending formal rehabilitation at the hospital, whereas patients following usual care declined significantly during this time [9]. New to the present study, is that the patients did only receive one hour of instruction in how to do the training, and no actual rehabilitation, before discharge from the hospital. This study therefore challenges the often met criticism to high intensity training as a non-feasible form of exercise for cardiac patients.

## Supporting Information

Checklist S1 CONSORT Checklist.
(DOCX)

Protocol S1 Trial Protocol.
(DOCM)
