# Supplementary material for: Home-Based Aerobic Interval Training Improves Peak Oxygen Uptake Equal to Residential Cardiac Rehabilitation: A Randomized, Controlled Trial
Source: PLoS One. 2012 Jul 18;7(7):e41199. doi: 10.1371/journal.pone.0041199 (PMC3399826; doi:10.1371/journal.pone.0041199)
Supplement: Protocol S1 — Trial Protocol. (DOCM) [file pone.0041199.s002.doc]

# Effective rehabilitation after coronary artery bypass surgery: Protocol

#### Background

# According to meta-analyses, cardiac rehabilitation that includes exercise training give a 20-31% reduction in mortality (O’Connor et al. 1989; Joliffe et al. 2000). Exercise training has also proven to reduce angina pectoris, to improve physical fitness and general health (Froelicher et al. 1984; Schuler et al. 1992; Niebauer et al. 1997; Lindsay et al. 2003). In Norway, both home based and institutional cardiac rehabilitation exist, and presently we do not know which of these rehabilitation modes that is most effective. Home-based rehabilitation is a kind of rehabilitation where the patients receive guidelines about how to exercise on their own, often with some kind of surveillance. Earlier studies have shown good adherence and improvements in physical capacity and health related quality of life equal to and also superior to patients following more organized cardiac rehabilitation (Marchionni et al, 2003; Kodis et al., 2001; Smith et al., 2004).

# It has been shown that a reduced physical capacity is the most important predictor of mortality in middle aged men with and without coronary heart disease (Myers et al. 2002; Kavanagh et al 2002). After exercise training, improvements are found in stroke volume, cardiac contractility, and left ventricular ejection fraction and volum, and also increased fibre areal, capillary density and enzymatic activity in skeletal muscle (Shephard & Balady 1999; Ades 2001). Several mechanisms have been suggested to explain the improved myocardial perfusion (Scheel et al. 1981; Cohen et al. 1982; Ernst 1987; Franklin et al. 1991; Schuler et al. 1992; Niebauer et al. 1995; Belardinelli et al. 2001; Gielen et al., 2001; Reinhart et al. 1998), butt hese mechanisms are probably not sufficient explainations to the positive effects of regular exercise training. The effect is probably even greater on another important blood flow regulation mechanism, that is the endothelial function (Furchgott et al. 1980; Ludmer et al. 1986; Palmer et al. 1987; Ross 1993).

# Aim

The aims of this study will be to evaluate different kinds of cardiac rehabilitation for coronary artery bypass surgery patients. The primary aim is to compare the effects of policlinical, institutional, and home-based cardiac rehabilitation on physical capacity, endothelial function and health related quality of life. We hope to answer the following:

- Is there a difference in the changes in physical capacity, health related quality of life, and endothelial function between patients attending policlinical and institutional rehabilitation?
- Is there a difference in the changes in physical capacity, health related quality of life, and endothelial function between patients attending home-based and institutional rehabilitation?

In advance, we have a hypotheses that institutional rehabilitation will improve physical capacity, health related quality of life, and endothelial function more than home-based and policlinical rehabilitation.

# Methods

# Participants

Patients who have undergone coronary artery bypass surgery at the St.Elisabeth Clinic and the Feiring Heart Clinic will be asked to participate in the study. Patients will be randomly allocated to two groups. At the St.Elisabeth Clinic, these will be institutional rehabilitation at Røros Cardiac Rehabilitation Centre or policlinical rehabilitation at the St.Olav Hospital. At the Feiring Heart Clinic, the two groups will be institutional rehabilitation at the Feiring Heart Clinic and home-based rehabilitation. The rehabilitation will start 4-8 weeks after the operation. The patients will be randomized after the operation, while they are still at the hospitals, after they have agreed to participate in the study. The randomization will be done by the technicians at the unit of Applied Clinical Research at the Norwegian University of Science and Technology, using a computer randomization code with block randomization.

According to the staff at the two hospitals, most patients have a clear opition about what kind of rehabilitation they wish to participate in after the surgery. To ensure that we will have patients included in the study, we will offer the patients allocated to policlinical rehabilitation or home-based rehabilitation to attend an institutional rehabilitation program after ending the 6 months follow-up period of the study.

With a statistical power of 0.8 (p<0.05) and an expected group difference in improvement in peak oxygen uptake of 3,0 mL/kg/min (SD 4,0), we will have to include 60 patients in total. We therefore aim to include 60-70 patients, with half of them at each site.

Patients included at the St.Elisabeth Clinic will have to live in or nearby Trondheim, making it possible to come for policlinical exercise training twice weekly at the hospital.

# Exclusion criteria

- Physical limitation not caused by the coronary heart disease
- Left ventricual ejection fraction < 30%
- Hemodynamical significant valve defect (> NYHA klasse II) or congenital heart disease
- Non-cardiac condition that may affect mortality negatively during the study period
- Reasons making it likely that the subject will not complete the study
- Kidney failure (kreatinin > 140)
- Lung disease
- Planned surgery during the project period
- Pregnancy
- Drug abuse

# Tests

St.Elisabeth: All assessments for patients at the St.Elisabeth Clinic will be done at the Department of Circulation and Medical Imaging at the Medical Faculty of the Norwegian University of Science and Technology. The assessements will be done before starting rehabilitation (baseline), after four weeks (end of institutional rehabilitation), at 12 weeks (end of policlinical rehabilitation), and at six months of follow-up.

The Feiring Heart Clinic: For patients at the Feiring Heart Clinic all assessments will be done at the Feiring Heart Clinic. The patients will be tested 4-8 weeks after the operation (baseline) and after 6 months.

All measurements of peak oxygen uptake will be done at treadmills. Before the test starts, the pasients will be given a brief oral explaination of the test. The protocol starts with a warming-up period of 10 minutes. The speed of the treadmill will be 3-6 km/h and the elevation starts at 0%. The load will increase every 1-2 minute, individually adjusted to the test person so that the test will last 8-12 minutes after the warm-up period. The test will stop when the patients no longer can continue due to exhaustion or to ischemic symptoms. After ending the test, the patients will be asked to rate their perceived exhaustion according to the Borg scale (Borg et al. 1981). Oxygen uptake and heart rate will be measured continuously. The oxygen uptake will be measured every tenth second. Peak oxygen uptake (VO_2peak_ ) will be registered as the average of the three highest measurements. At the peak, we will register the fall in heart rate during the first minute. All tests will be done with electrocardiogram monitoring and with a physician present.

Questionnaires on health related quality of life will be SF-36 (Ware et al. 1993) and MacNew (Lim et al. 1993).

Endothelial function and blood samples will be measured after an overnight fast. Patients will rest for 15 min on the examination bench before the measurements start. Endothelial function will be measured as flow-mediated dilatation of the brachial artery. We will measure this above the antecubital fossa according to the guidelines by Coretti et al. (2002). The artery diameter and blood flow will be registered before the blood flow will be stopped by a standard blood pressure cuff for 5 minutes, with a pressure of 250 mmHg. Then the pressure is released and the diameter and blood flow is measured at 1, 2, 3, 4, and 5 minutes after the release.

Blod samples will be taken to measure total cholesterol, HDL, LDL, triglycerides, and markers of endothelial function (endotelin, ICAM, VCAM og eNOS).

# Intervention

St. Elisabeth: Both the policlinical and the institutional rehabilitation may be regarded as standard care for these patients.

Patients included in the policlinical rehabilitaton will receive 12 weeks policlinical rehabilitation with twice weekly exercise training for 60 min. Exercise training will take place at the St.Olavs Hospital and be lead by a physiotherapist. In advance of the first session, the patients will get an introduction about the aim and the content of the exercise sessions. In addition, patients and their next of kind will be offered to attend a meeting about the cardiac rehabilitation. The main aim of the exercise sessions will be to improve aerob endurance. There will also be some strength training, and flexibility training. The intensity during exercise will be set by the use of the Borg scale (Borg et al. 1981) and there will be no objective control of individual exercise intensity.

Patients included in institutional rehabilitation will attend a 4 week cardiac rehabilitation at the Røros Cardiac Rehabilitation Centre. They will be followed by an interdisciplinary team with a physician, physiotherapists, nurses, nutritionist, and psychologist. The patients will exercise one or two times each day for five days per week. The main aim of the exercise training will be to improve aerob endurance. The intensity will be set using the Borg scale (Borg et al. 1981) and there will be no objective controll of individual exercise intensity. Patients will receive education on psychologial reactions to cardiac disease, cardiac anatomi, physiology, and pathology, medicaments, first aid and more. The education will be 3-4 hours per week.

The Feiring Heart Clinic: The institutional rehabilitation offered at The Feiring Heart Clinic will be equal to the one at the Røros Cardiac Rehabilitation Centre (as described above).

Patients randomized to home-based rehabilitation will get advice about exercise training, how to quit smoking and diet when they are at the Feiring Heart Clinic for baseline assessments. They will receive no further surveillance from the clinic during the study period of six months, but are offered to contact the clinic if they have any questions about the rehabilitation during this time period.

**Referances:**

Ades PA (2001) Cardiac rehabilitation and secondary prevention of coronary heart disease *New England Journal of Medicine.* 345, 892-902

Anderson TJ, Uehata A, Gerhard MD, Meredith IT, Knab S, Delagrange D, Lieberman EH, Ganz P, Creager MA, Yeung AC, Selwyn AP. (1995). Close relation of endothelial function in the human coronary and peripheral circulations. *J Am Coll Cardiol*, 6, 1235-1241

Belardinelli R, Paolini I, Cianci G, Piva R, Georgiou D, Purcaro A (2001). Exercise training intervention after coronary angioplasty: the ETICA trial. *J Am Coll Cardiol*, 37, 1891-1900

Borg G, Holmgren A, Lindblad I (1981). Quantitative evaluarions of chest pain. *Acta Med Scand (Suppl)*, 644, 43-45

Brooks GA, Fahey TD, White TP, Baldwin KM (2000). *Exercise Physiology. Human bioenergetics and its applications. (3^rd^ ed.).* Mayfield Publishing Company

Cohen MV, Yipintsoi T, Scheuer J (1982). Coronary collateral stimulation by exercise in dogs with stenotic coronary arteries.  *J Appl Physiol*, 52, 664-671

Coretti MC, Anderson TJ, Benjamin EJ, Clermajer D, Charbonneau F, Creager MA, Deanfield J, Drexler H, Gerhard-Herman M, Herrington D, Vallance P, Vita J, Vogel R (2002). Gudielines for the ultrasound assessment of endothelial-dependent flow mediated vasodiation of the brachial artery: a report of the International Brachial Artery Reactibity Task Force. *J Am Coll Cardiol,* 39, 257-265

Detry JR, Vierendeel IA, Vanbutsele RJ, Robert AR (2001). Early short-term intensive cardiac rehabiliteation induces positive results as long av one year after the acute coronary event: a prosepctive one-year controlled study. J Cardiovasc Risk, 8, 355-61

Ernst E (1987). Influence of regular physical activity on blood rheology. *Eur Heart J,* 8, 59-62

Fletcher GF, Balady G, Blair SN, Blumenthal J, Caspersen C, Chaitman B, Epstein S, Sivarjan Froelicher ES, Froelicher VF, Pina IL, Pollock ML (1996). Statement on exercise: benefits and recommendations for physical activity programs for all Americans. A statement for health professionals by the Committee on Exercise and Cardiac Rehabilitation of the Council on Clinical Cardiology, American Heart Association. *Circulation*, 94, 857-862

Fletcher GF (1997). How to miplement physical activity in primary and secondary prevention. A statement for healthcare-professionals from the Task Force on Risk-reduction, Aamerican Heart Association, *Circulation*, 96, 355-357

Fletcher GF, Balady GJ, Amserdam EA, Chaitman B, Eckel R, Fleg J, Froelicher VF, Leon AS, Pina IL, Rodney R, Simons-Morton DA, Williams MA, Bazzarre T (2001). A statement for healthcare professionals from the American Heart Association. *Circulation*, 104, 1696-1740

Franklin BA (1991). Exercise training and coronary collateral circulation. *Med Sci Sports Exerc* 104, 1694-1740

Froelicher V, Jensen D, Genter F, Sullivan M, McKrinan MD, Witztum K, Scharf J, Strong ML, Ashburn W (1984). A randomized trial of exercise training in patients with coronary heart disease. *JAMA*, 252, 1291-1297

Furchgott RF, Zawadzki JV (1980). The obligatory role of endothelial cells in the relaxation of arterial smooth muscle by acetylcholine. *Nature*, 288, 373-376

Gielen S, Schuler G, Hambrecht R (2001). Exercise training in coronary artery disease and coronary vasomotion. *Circulation*, 103, 1-6

Hauer K, Niebauer J, Weiss C, Marburger C, Hambrecht R, Schlierf G, Schuler G, Zimmermann R, Kubler W (2000). Myocardial ischemia during physical exercise in patients with stable coronary artery disease: predictability and prevention. *Int J Cardiol* 75, 179-186

Jolliffe JA, Rees K, Taylor RS, Thompson D, Oldridge N, Ebrahim S (2000). Exercise-based rehabilitation for coronary heart disease. *Cochrane Database Syst Rev* 4, CD001800

Kavanagh T, Mertens DJ, Hamm LF, Beyene J, Kennedy J, Corey P & Shephard RJ (2002). Prediction of long-term progrnosis in 12 169 men referred for cardiac rehabilitation. *Circulation*, 106, 666-671

Kiebzak GM, Pierson LM, Campbell M & Cook JW (2002). Use of the SF36 general health status survey to document heart related quality of life in patients with coronary artery disease. The effect of disease and response to coronary artery bypass graft surgery. *Heart Lung*, 31, 207-213

Kodis, J., Smith, K, Arthur, H., Daniels, C., Suskin, N., McKelvie, R. (2001). Changes in exercise capacity and lipids after clinic versus home-based aerobic training in coronary artery bypass graft surgery patients. *Journal of Cardiopulmonary Rehabilitation,* 21(1), 31-36

Lee IM, Sesso HD, Oguma Y, Paffenbarger RS Jr. (2003). Relative intensity of physical activity and risk of coronary heart disease. *Circulation.* 107, 1110-6.

Lim LL, Valenti LA, Knapp JC, Dobson AJ, Plotnikoff R, Higginbotham B, Heller RF (1993). A self-administered quality-of-life questionnaire after acute myocardial infarction. *J Clin Epidemiol,* 46, 1249-56

Lindsay GM, Hanlon WP, Smith LN, Belcher PR (2003). Experience of cardiac rehabilitation after coronary artery surgery: effects on health and risk factors. *Int J Cardiol*, 87, 67-73

Ludmer PL, Selwyn AP, Shook TL, Wayne RP, Mudge GH, Alexander RW, Ganz P (1986). Paradoxical vasoconstriction induced by acetylcholine in atherosclerotic coronary arteries. *N Engl J Med*, 315, 1046-1051

Manson JE, Greenland P, LaCroix AZ, Stefanick ML, Mouton CP, Oberman A, Perri MG, Sheps DS, Pettinger MB, Siscovick DS (2002). Walking compared with vigorous exercise for the prevention of cardiovascular events in women. *N Engl J Med,* 347, 716-25

Marchionni, N, Fattirolli, F, Fumagalli, S., Oldridge, N., Del Lungo, F., Morosi, L., Burgisser, C., Masotti, G. (2003). Improved exercise tolerance and quality of life with cardiac rehabilitation of older patients after myocardial infarction. Results of a randomized, controlled trial. *Circulation, 107, 2201-2206*

Niebauer J, Hambrecht R, Marburger C, Hauer K, Velich T, von Hodenberg E, Schlierf G, Kubler W, Schuler G (1995). *Am J Cardiol*, 76, 771-775

Niebauer J, Hambrecht E, Velich T, Hauer K, Marburger C, Kalberer B, Weiss C, von Hodenberg E, Schlierf G, Schuler G, Zimmermann R, Kubler W (1997). Attenuated progression of coronary artery disease after 6 years of multifactorial risk intervention: role of physical exercise. *Circulation*, 96, 2534-2541

O’Connor GT, Buring JE, Yusuf S, Goldhaber SZ, Omestead EM, Paffenbarger RS jr, Hennekens CH (1989). An overview of randomized rials of rehabilitation with exercise after myocardial infarction. *Circulation*  80, 234-244

Palmer RM, Ferrige AG, Moncada S (1987). Nitric oxide release accounts for the biological activity of endothelium-derived relazing factor. *Nature,* 327, 524-526

Raitakari & Celermajer (2000)

Raitakari OT, Celermajer DS (2000). Testing for endothelial dysfunction. Ann Med, 32, 293-304

Reinhardt WH, Dziekan G, Goebbels U, Myers J, Dubach P (1998). Influence of exercise training on blood viscosity in patients with coronary artery disease and impaired left bventricular function. *Am Heart J* 135, 379-382

Ross R (1993). The parhogenesis of atherosclerosis: a perspective for the 1990s. *Nature* 362, 801-809

Scheel KW, Ingram LA, Wilson JL (1981). Effects of exercise on the coronary and collateral vasculature of beagles with and without coronary occlusion.  *Circ Res* 48, 523-530

Schuler G, Hambrecht R, Sclierf G, Grunze M, Methfessel S, Hauer K, Kubler W (1992). Myocardial perfusion and regression of coronary artery disease in patiens on a regime of intensive physical exercise and low fat diet. *J Am Coll Cardiol* 19, 34-42

Shephard RJ & Balady GJ (1999). Exercise as cardiovascular therapy. *Circulation,*  99, 963-972

Smith, K., Arthur, H., McKelvie, R., Kodis, J. (2004). Differences in sustainability of exercise and health-related quality of life outcomes following home or hospital-based cardiac rehabilitation. *European Journal of Cardiovascular Prevention and Rehabilitation,* 11, 313-319

Swain DP & Franklin BA (2002). Is there a threshold intensity for aerobic training in cardiac patients?

*Med Sci Sports Exerc*, 34, 1071-5.

Takase B, Uehata A, Akima T, Nagai T, Nishioka T, Hamabe A, Satomura K, Ohsuzu F, Kurita A. (1998). Endothelium-dependent flow-mediated vasodilation in coronary and brachial arteries in suspected coronary artery disease. *Am J Cardiol*. 82, 1535-9

Takeyama, J, Itoh H, Kato M, Watanabe H, Nagayama M, Katagiri (2000). Effects of physical training on the recovery of the autonomic nervous activity during exercise after coronary artery bypass grafting. Effects of physical training after CABG. *Jpn Circ J*, 64, 809-813

Tanasescu M, Leitzmann MF, Rimm EB, Willett WC, Stampfer MJ, Hu FB (2000). Exercise type and intensity in relation to coronary heart disease in men. *JAMA*, 288, 1994-2000

Ware J, Snow KK, Kosinski M, Gandek B (1993). *SF-36 Health survey: Manual and interpretation guide.* Boston, Massachusetts: The Health Institue, New England Medical Centre

Wenger, HA & Bell, GJ (1986). The interactions of intensity, frequency and duration of exercise training in altering cardiorespiratory fitness. *Sports Med*, 3, 346-56.
